# Statistical data on the physical and mechanical properties of fibre reinforced alkali activated uncalcined earth based composite

**DOI:** 10.1016/j.dib.2019.104839

**Published:** 2019-11-18

**Authors:** Emeso B. Ojo, Kabirat O. Bello, Ronaldo S. Teixeira, Peter A. Onwualu, Holmer Savastano

**Affiliations:** a African University of Science & Technology(AUST) Materials Science & Engineering Dept., Abuja, Nigeria; b Nigerian Building & Road Research Institute (NBRRI), Building Research Dept., Abuja, Nigeria; c São Paulo State University (UNESP), Materials &Technology Department, São Paulo, Brazil

**Keywords:** Earth based composites, Fibre reinforced composites, Alkali activation, Uncalcined earth composite, Modulus of rupture

## Abstract

This article presents statistical data on the reinforcing effect of three different fibres (sisal, eucalyptus pulp and polypropylene) on the physical and mechanical properties of an alkali activated natural soil produced using extrusion technique for the development of earth-based building materials. The experimental testing program involved characterisation of composite mixtures including a plain unreinforced stabilised matrix (which was plain soil mixed with alkali activator solution) as well as composite mixtures incorporating 3 volume fractions of fibres (0.5, 1.0 & 2.0 vol.%) of each fibre type. Composites were tested to evaluate physical properties (density and water absorption) and flexural response under 4-point loading in both dry and saturated conditions. The obtained values were statistically analyzed using one-way analysis of variance (ANOVA), followed by Tukey multiple comparison tests to ascertain the effect of the reinforcing fibres on the physical and mechanical properties of the composites. Results obtained show unique reinforcing effects of the different fibre types in the alkali activated matrices and the sensitivity of the earth based matrix to variations in fibre volume fraction. This data article is related to “Effects of Fibre Reinforcements on Properties of Extruded Alkali Activated Earthen Building Materials” [1].

**Table:** Specifications Table

Subject	Materials Science
Specific subject area	Fibre reinforced Earthen Building Materials
Type of data	Tables
How data were acquired	Bulk Density (ASTM C948) Water absorption (ASTM C948) Four-point bending test (ASTM D1635-00)
Data format	Raw Statistically Analysed
Parameters for data collection	Composite mixtures including; a plain unreinforced stabilised matrix (which was plain soil mixed with alkali activator solution) as well as mixtures incorporating 3 volume fractions of fibres (0.5, 1.0 & 2.0 vol.%) for three different fibres was developed and tested after 14 days
Description of data collection	ANOVA and Tukey's HSD Comparison analysis on the Physical properties (density and water absorption) and flexural response (MOR) under 4-point loading in dry and saturated conditions.
Data source location	Research Nucleus on Materials for Biosystems (NAP BioSMat), Laboratory of Construction and Ambience, Campus Fernando Costa – University of São Paulo Pirassununga/SP, 225 Duque de Caxias Norte Avenue Brazil, 13635-900
Data accessibility	Data are presented in this article
Related research article	Ojo, E. B., Bello, K. O., Mustapha, K., Teixeira, R. S., Santos, S. F., & Savastano Jr, H. (2019). Effects of fibre reinforcements on properties of extruded alkali activated earthen building materials. Construction and Building Materials, 227, 116778. https://doi.org/10.1016/j.conbuildmat.2019.116778

**Table:** 

**Value of the Data** • The data shows unique reinforcing effects of varying fibre types on alkali activated earth based composites which is useful in the selection of appropriate fibres for reinforcement of earth based matrices for various applications. • The data shows sensitivity of properties of earth based matrix to variations in fibre contents which is useful in the selection in suitable fibre volume fractions for reinforcement. • These data may guide other combinations of fibres that may enhance properties. • These data present insights on reinforcing mechanisms for varying fibre types

## 1 Data

The details of the experimental data used for the statistical analysis presented in this article are presented in Table 1. The reinforcing effect of the fibres (sisal, eucalyptus pulp and polypropylene) on the physical and mechanical properties of the earth-based composites were statistically analyzed using one-way ANOVA, followed by Tukey multiple comparison tests. The ANOVA and Tukey's HSD Comparison of mean values of densities (Table 2, Table 3), water absorption (Table 4, Table 5), flexural properties in Dry Condition (Table 6, Table 7) and flexural properties in saturated Condition (Table 8, Table 9) for different fibre types and contents are presented. The significance level was set at 5%.

**Table 1:** Experimental dataset used in the statistical analysis.

Reinforcement type	Fibre Vol. %	Density kg/m^3^					
Plain Matrix	0	1.69	1.69	1.70	1.72	1.70	1.69
Sisal	0.5	1.73	1.73	1.73	1.74	1.75	1.77
	1	1.75	1.74	1.73	1.73	1.72	1.73
	2	1.71	1.73	1.70	1.69	1.67	1.71
E.Pulp	0.5	1.67	1.67	1.67	1.67	1.68	1.67
	1	1.73	1.69	1.71	1.70	1.71	1.70
	2	1.67	1.71	1.71	1.69	1.68	1.70
Polypropylene	0.5	1.61	1.68	1.68	1.69	1.68	1.69
	1	1.71	1.71	1.71	1.71	1.78	1.71
	**Fibre Vol. %**	**Water Absorption %**					
Plain Matrix	0	20.91	20.58	20.30	19.45	20.58	20.30
Sisal	0.5	19.56	19.60	19.43	19.16	19.15	18.08
	1	19.24	19.33	19.40	19.35	19.62	19.62
	2	20.04	19.68	20.04	20.48	20.14	20.14
E.Pulp	0.5	21.52	21.42	21.25	21.13	21.09	21.27
	1	19.57	20.59	20.04	20.22	20.04	20.07
	2	21.26	19.81	19.70	20.53	20.82	19.98
Polypropylene	0.5	27.10	21.58	21.61	21.25	21.67	21.27
	1	20.16	20.27	20.31	20.51	15.15	15.15
	**Fibre Vol. %**	**Modulus of Rupture (Dry condition) MPa**					
Plain Matrix	0	2.62	3.58	3.49	2.80	3.01	
Sisal	0.5	6.03	4.67	5.34	6.35	5.74	
	1	4.94	5.62	5.72	5.11	6.78	
	2	4.83	4.46	5.50	5.35	4.97	
E.Pulp	0.5	3.18	3.76	2.08	1.80	2.90	
	1	4.25	4.01	4.98	4.93	4.55	
	2	3.83	3.39	3.83	3.50	3.30	
Polypropylene	0.5	3.60	4.41	3.32	3.03	2.84	
	1	4.26	2.44	3.79	3.45	3.92	
	**Fibre Vol. %**	**Modulus of Rupture (Saturated Condition) MPa**					
Plain Matrix	0	0.32	0.34	0.65	0.38		
Sisal	0.5	0.60	1.00	1.03	0.89	1.20	
	1	1.30	1.03	0.98	1.30	1.08	
	2	0.86	0.95	0.85	0.96	0.89	
E.Pulp	0.5	0.60	1.00	1.03	0.89	1.20	
	1	1.30	1.03	0.98	1.30	1.08	
	2	0.86	0.95	0.85	0.96	0.89	
Polypropylene	0.5	0.65	0.67	0.75	0.95	0.74	
	1	1.61	1.27	0.93	1.10	1.33

**Table 2:** Results of 5% ANOVA tests on densities for various fibre reinforcements and contents.

		df	F	Sig.
Sisal	Between groups	3	15.17	2.2 × 10^−5^
	Within groups	20		
Eucalyptus pulp	Between groups	3	8.06	0.001
	Within groups	20		
Polypropylene	Between groups	2	5.11	0.02
	Within groups	15

**Table 3:** Results of Tukey's HSD Comparison of mean densities for different fibre types and contents.

Fibre vol.%	Sisal			Eucalyptus pulp			Polypropylene	
	0	0.5	1.0	0	0.5	1.0	0	0.5
0	–	–	–	–	–	–	–	–
0.5	1.635E-4*	–	–	0.007*	–	–	0.193	–
1.0	0.002*	0.672	–	0.743	7.789E-4*	–	0.389	0.016*
2.0	0.997	2.485E-4*	0.003*	0.845	0.046*	0.289		

Note: Numbers with asterix (*) indicate statistically significant differences at 0.05 level.

**Table 4:** Results of 5% ANOVA tests on of water absorption for different fibre types and contents.

		df	F	Sig.
Sisal	Between groups	3	11.22	1.56 × 10^−4^
	Within groups	20		
Eucalyptus pulp	Between groups	3	8.52	7.62 × 10^−4^
	Within groups	20		
Polypropylene	Between groups	2	5.21	0.02
	Within groups	15

**Table 5:** Results of Tukey's HSD Comparison of mean values of water absorption for different fibre types and contents.

Fibre vol.%	Sisal			Eucalyptus pulp			Polypropylene	
	0	0.5	1.0	0	0.5	1.0	0	0.5
0	–	–	–	–	–	–	–	–
0.5	3.137E-4*	–	–	0.008*	–	–	0.224	–
1.0	0.004*	0.675	–	0.729	7.307E-4*	–	0.325	0.015*
2.0	0.674	0.004*	0.048*	1.000	0.007*	0.732		

Note: Numbers with asterix (*) indicate statistically significant differences at 0.05 level.

**Table 6:** Results of 5% ANOVA tests on of MOR for different fibre types and contents in the dry condition.

		df	F	Sig.
Sisal	Between groups	3	13.294	4.02 × 10^−4^
	Within groups	16		
Eucalyptus pulp	Between groups	3	5.79	0.02
	Within groups	16		
Polypropylene	Between groups	2	0.35	0.72
	Within groups	12

**Table 7:** Results of Tukey's HSD Comparison of mean values of MOR for different fibre types and contents in the dry condition.

Fibre vol.%	Sisal			Eucalyptus pulp			Polypropylene	
	0	0.5	1.0	0	0.5	1.0	0	0.5
0	–	–	–	–	–	–	–	–
0.5	3.94E-4*	–	–	0.929	–	–	0.988	–
1.0	0.001*	0.909	–	0.019*	0.09	–	0.757	0.768
2.0	0.002*	0.404	0.792	0.617	0.952	0.128		

Note: Numbers with asterix (*) indicate statistically significant differences at 0.05 level.

**Table 8:** Results of 5% ANOVA tests on of MOR for different fibre types and contents in the saturated condition.

		df	F	Sig.
Sisal	Between groups	3	16.71	3.47 × 10^−5^
	Within groups	16		
Eucalyptus pulp	Between groups	3	13.23	2.26 × 10^−4^
	Within groups	16		
Polypropylene	Between groups	2	22.09	1.41 × 10^−4^
	Within groups	12

**Table 9:** Results of Tukey's HSD Comparison of mean values of MOR for different fibre types and contents in the saturated condition.

Fibre vol.%	Sisal			Eucalyptus pulp			Polypropylene	
	0	0.5	1.0	0	0.5	1.0	0	0.5
0	–	–	–	–	–	–	–	–
0.5	5.70E-4*	–	–	0.543	–	–	0.059	–
1.0	1.79E-5*	0.148	–	0.022*	0.269	–	1.15E-4*	0.004*
2.0	0.002*	0.989	0.102	2.01E-4*	0.003*	0.07		

Note: Numbers with asterix (*) indicate statistically significant differences at 0.05 level.

## 2 Experimental design, materials, and methods

### 2.1 Characterisation of materials

#### 2.1.1 Soil

Soil used in this study was supplied by Top Telha Ceramic Tile Company in Leme, São Paulo State, Brazil. Particle size distribution of the soil was determined by mechanical sieving and hydrometer sedimentation in accordance with BS 1377:2. The elemental composition of the soil and X-Ray Diffraction (XRD) performed using a Rigaku MiniFlex 600 with range 10–70° (2θ) at a rate of 0.02°/min was determined. Results are presented in Ref. [1].

#### 2.1.2 Alkali activator solution

Alkaline activator was prepared using a solution of sodium hydroxide and sodium silicate. Sodium hydroxide (NaOH) used was of caustic soda pellets with 97% purity whilst sodium silicate powder was used and both chemicals were produced by Dinâmica Química Contemporânea Ltd, Brazil. The weight ratio of sodium hydroxide/soil and sodium silicate/soil was 3% and 0.75% respectively. Alkaline activator solution was prepared by dissolving requisite quantities of pellets/powders in deionized water and allowing to cool for 24 h [1].

#### 2.1.3 Fibres

Two ligno-cellulosic fibres (sisal, Eucalyptus pulp microfibers) and one synthetic fibre (polypropylene) were used in this study. Sisal (*Agave sisalana*) fibres were sorted and manually cut to average lengths of 10 mm. Unbleached hardwood kraft pulp extracted from *Eucalyptus urophylla*was obtained from the paper manufacturing process and produced in São Paulo, Brazil was used in this study. Polypropylene fibre produced by Saint-Gobain, Brazil was selected in this study for the basis of comparison with the vegetable fibres. Properties of fibres expressed as average values ± standard deviation are presented in Ref. [1].

### 2.2 Mixtures and preparation of specimens

Ten different mixtures were prepared for this study; a plain unreinforced matrix (which was plain soil mixed with alkali activator solution) as well as 9 other mixtures incorporating 3 volume fractions of fibres (0.5, 1.0 & 2.0 vol.%) for the three different fibres. For the reinforced composites, soil was replaced with fibres corresponding to required volume fraction.

In the preparation of unreinforced composites, soil and alkali activator solution were homogenized using a high energy intensive Eirich mixer (capacity 10 L) for 5 min at high speed. Sisal and polypropylene fibre-soil composites were produced by first homogenising soil and the respective fibres in the dry state for 5 min at high speed before the introduction of the alkali activator solution with additional mixing for 5 min. In the case of the pulp-soil mixture, pulp fibres were dispersed in the alkaline solution using a magnetic stirrer for 10 min before transferring it to the soil and homogenising for 5 min in the mixer.

Following homogenization, fibre-soil mixtures were transferred to a laboratory extruder (Gelenski, model MVIG-05 with a cross section die width/height ratio of 3.3 and operating at a linear speed of approximately 4 mm/s. Plates of 200 mm × 50 mm × 15 mm were produced and samples were cured initially at elevated temperature of 105 °C for 5 h and left in the laboratory at room temperature of (24 ± 2)°C and relative humidity (RH) of 60% for 14 days [2].

### 2.3 Physical tests

After curing, a minimum of six samples were subjected to physical tests: apparent void volume bulk density and water absorption were determined according to ASTM C948:

(i) $$BulkDensity\left(g/cm^{3}\right)=\left(\frac{M_{dry}}{M_{sat}-M_{i}}\right)\times \rho$$

(ii) $$WaterAbsorption\left(\%\right)=\left(\frac{M_{sat}-M_{dry}}{M_{dry}}\right)\times 100$$

where M_sat_ is the saturated specimen's mass with a dry surface, M_dry_ is the dry specimen's mass after 24 hours at 105 °C, M_i_ is the specimen's mass immersed in water and ρ is the bulk density of water (g/cm^3^).

### 2.4 Mechanical test

Flexural tests were performed on a minimum of six specimens using a universal testing machine Emic DL-30000 equipped with 5 kN load cell. A four-point bending configuration was adopted using a displacement control testing procedure at a rate of 1 mm/min. Composites were tested in dry condition where samples were subjected to 60 °C for 24 h prior to testing; as well as saturated condition were another set of samples were fully immersed in water for 24 h, prior to testing. Modulus of Rupture (MOR) was measured [1].

(iii) $$MOR=\frac{Fl}{bd^{2}}$$

where *F* is the maximum load, *l* is the major span length, *b* and *d* are the sample's respective width and depth.

### 2.5 Statistical analysis

To aid in the interpretation of results, statistical analysis was performed using ANOVA analysis in order to ascertain the effect of the reinforcing fibres on the physical and mechanical properties of the composites. One-way ANOVA tests was used to determine if differences existed between population means obtained at varying fibre volumes for a specific fibre reinforcement. The procedure tested the null hypothesis (H_o_) that the average results at all fibre dosages are equal (suggesting that the incorporation of fibres had no effect on the performance) against the alternative hypothesis (H_a_) that at least one average result was different i.e

(iv) $$H_{0}:\mu_{1}=\mu_{2}=\mu_{3}=\mu_{4}$$

$$H_{a}:Atleastonemeanisdifferentfromtheothers$$

where μ_1_, μ_2_, μ_3_ and μ_4_ are population means corresponding to different fibre volume fractions. A significance level of 5% was adopted for this study indicating that if the p-value of a statistical test was less than 0.05, at least one of the population means was statistically different from the others and the null hypothesis (H_o_)was rejected. If H_o_ was rejected, a post-hoc test (Tukey's Honestly Significance Difference (HSD) test) was performed to determine which means were statistically different from each other. This test was performed to ascertain if varying fibre contents had any statistical effect on the performance of the composites.
